# Issues of Unequal Access to Public Health in India

**DOI:** 10.3389/fpubh.2015.00245

**Published:** 2015-10-27

**Authors:** Debasis Barik, Amit Thorat

**Affiliations:** ^1^National Council of Applied Economic Research, New Delhi, India

## Introduction

Health in India is a state subject. Although the central government shares a significant part in establishing health care infrastructure, each of the Indian states determines their priorities for health care financing, and provides services to the population. India’s 12th plan document^1^ promises to build upon the initiatives that were taken in the 11th plan and expand the reach and coverage of health care to achieve the long-term objective of “universal health care.”

Irrespective of the ability to pay, people in India increasingly seek private health care even for minor illnesses like cold, fever, and diarrhea. Private health care in India, however, is not only expensive but also suffers severely from a lack of trained and skilled manpower as compared to the public sector (2). Access to health care facilities is significantly urban biased. So, people living in the rural areas face the additional handicap of such a situation and they form a disproportionately larger share of the unhealthy population.

With respect to access to health care, the 12th plan document states that “Barriers to access would be recognized and overcome especially for the disadvantaged and those living far from facilities.” The document goes on to mention that “… the SC and ST,^2^ the particularly vulnerable tribal groups, the de-notified^3^ and nomadic tribes, the Musahars^4^ and the internally displaced must be given special attention while making provisions for, setting up and renovating sub-centers and anganwadis^5^.”

These groups need special attention as they not only suffer from unequal and lower access but also produce the worst health outcomes in the country. This is primarily because these groups have been traditionally excluded and discriminated, and therefore suffer from high incidences of poverty and low levels of education (health care awareness), among other disadvantages, which have made their access to public health care tougher. While the public health care system required to have ensured better care and treatment for these marginalized communities, evidence shows that access remains the lowest among these population group.

In this paper, we focus on the issues of unequal access to health care in India by rural–urban residence, economic status, and caste/religion identity.

## Access to Health Care

Poor housing condition, unsafe drinking water, lack of sanitation, use of biomass fuels, exposure to environmental odds as a part of the livelihood among the marginal population group often increase the risk of numerous health problems. Desai et al. (3) noted a very high prevalence of minor ailments like cough, fever, diarrhea. (124 per 1,000 individuals) among Indian population. The minor illnesses despite being short term in nature cause substantial time loss from usual activities. The prevalence of these minor ailments is seen to vary substantially by socio-economic conditions of households. These are more prevalent among the poor and the uneducated population and those who belong to the scheduled tribe community. The prevalence seems to reduce with the improvement in living conditions. However, everybody benefits from living in a metro city, regardless of their social position.

Treatment rates across groups do not show much variation for minor illnesses. Minor illnesses do not require much laboratory test and people in rural areas prefer to go to a private provider for such types of illnesses due to easy availability and greater convenience. The major share of the cost of minor illnesses is the doctors’ fees and medicine. But, disparity in health care seeking between various socio-economic groups becomes prominent in case of major illnesses like hypertension, heart diseases, diabetes etc. Major illnesses are long term in nature and subject to a number of diagnostic tests. A sizeable proportion of major illnesses in rural areas remain untreated mainly due to unavailability of diagnostic facilities in the local vicinity. Desai et al. (3) have shown that only 3% of the major illnesses in metro areas remain untreated, whereas 12% of the same remain untreated in the less developed villages. Again, one-fifth of the diagnosed major illness among the scheduled tribes remain untreated. The tribal households are usually located in places, which have fewer health facilities and still rely on the traditional healers. A majority of these long-term major illnesses also remain undiagnosed amongst them. They need to go out of the villages, which are often isolated to avail treatment.

Access to health care is very much asymmetric between rural and urban India. While urban residents have a choice between public or private providers, the rural residents face far fewer choices. India has a very vast public health network with sub-centers working at the community level. The health sub-centers are manned mainly by bare foot health workers and work as a bridge between community and the primary health centers (PHC). PHC is the first contact point between village community and medical officer; meant to provide an integrated curative and preventive health care to the rural population with emphasis on preventive and promotive aspects of health care. Community health centers (CHC) are more equipped and acts mainly as a first referral unit with diagnostic facilities and a bunch of specialists. Since the recommendations of the Bhore Committee in 1946, a lot of emphasis has been put on the door step delivery of the health services. But, availability of any health facilities does not seem enough to attract people to the government facilities. Desai et al. (3) further noted that the possibility of visiting a government facility for minor illnesses reduce in the presence of any private facilities in the locality, but the reduction is much lesser for larger health care units like the CHC than the sub-centers.

## Cost of Treatment for Major and Minor Illnesses

The envisioned universal access to health care is far from achieving its goals. Over time, a lot of emphasis has been placed on the doorstep delivery of health services. However, the scheme-wise expenditure on India’s National Rural Health Mission (NRHM) during 11th Plan (2007–2012) on public health care expenditure reveals that a major share of the allocated resource on health was spent on family welfare program (90%), leaving a small segment (7.7%) for disease control (4).

Though investment in family welfare program is necessary, investment in disease control program cannot be ignored. Limited public health spending and higher emphasis on family planning services over time has resulted into a huge scarcity of resources to be spent on general health. A lot of public health facilities have been initiated in the outreach areas in the last decade, but due to unavailability of quality doctors and diagnostic facilities, people rush to the equally poor private facilities and end up spending more, almost all of which is out-of-pocket (OOP) expense.

## Impact of Medical Expenditure on Household Well-Being

Does the health expenditure cost the same to each household? This remains a major policy concern in many of the developing countries including India, where household OOP payment for health care is a significant part of the total health expenditure. The high OOP spending on health often leads to catastrophic level of spending for healthcare to many households and push them into poverty (5–7). The proportion of households facing catastrophic OOP health payments during 2004–2005, as measured by Ghosh (7) was 15.4% and the range varies as less as 3.5% in Assam to 32.4% in Kerala. Barik and Desai (8) measured the expenditure ratio (health expenditure as a percentage of income) on health care in India as 6% of the monthly average income, which is higher than the common benchmark of affordability (5%) in developing countries (9, 10). Moreover, this health burden is disproportionately distributed among various socio-economic groups. Poor households spent nearly 15% of their monthly income on healthcare compared to the richest households, who spend <1% of their income (Table 1).

**Table 1:** **Share of total household income spend on health care in India, 2004–2005**.

	Health care spending (%) on monthly household income		
	Any morbidity	Short term	Long term
**All India**	6.02	4.43	1.59
**Place of residence**			
Metro	1.13	0.67	0.46
Other urban	3.57	2.42	1.15
More developed village	7.73	5.72	2.01
Less developed village	6.87	5.18	1.69
**Income**			
Lowest quintile	14.53	11.15	3.38
Second quintile	4.53	3.27	1.26
Thirrd quintile	2.44	1.74	0.7
Fourth quintile	1.44	1.02	0.42
Top quintile	0.65	0.37	0.28
**Social groups**			
High caste Hindu	5.13	3.65	1.48
OBC	7.59	5.66	1.93
Dalit	5.32	4.06	1.26
Adivasi	3.88	2.78	1.1
Muslim	4.84	3.88	0.96
Other religion	9.19	4.36	4.83

*Barik and Desai (8), p. 57*.

As discussed above, the income share of the cost of treatment appears much higher on the socially and economically disadvantageous households. These higher health care cost often discourages them to avail treatment as reflected in case of major illnesses. More than two-thirds of the total health expenditure in India is met through household *OOP*. The coverage of health insurance is also very low among the Indians. Social insurance schemes contribute only 1.13% of the total health expenditure (11).

Besides availability and affordability, as discussed above, acceptability and adequacy are the two other important aspects of access to health care (12). A persistent negative attitude toward public health facilities in India has been recorded in a number of studies (13, 14). Das and Hammer (13) evaluated the quality of medical practices as a function of doctor’s competence in terms of knowledge of diseases and the practice of existing knowledge. They found that doctors in the public facilities are more qualified than the private doctors, but they use their knowledge less than what they should do in practice. Again, few studies have pointed out doctor’s absenteeism as the leading cause of people’s avoidance to government health facilities (15, 16). Complaints regarding long waiting hours, lack of privacy in the consultation room etc. are some common supply side constraints of public health system in developing countries including India (17, 18).

## Discussion

Even after more than 50 years of independence, health in India remains a luxury and only the rich can afford it. People visit equally poor private practitioners, ignoring the available public health units, and pay beyond their capacity. Quality health services, either public or private, with some government regulation, can help to improve the present scenario. The adivasi and the dalits are still away from the health equity and face more challenges than the others. Well-equipped health facilities in the vicinity and knowledge of disease conditions can improve the access of public health services. Rather than focusing on the doorstep services, well-equipped PHCs even can do better. A recent study by Goel and Khera (16) noted that provision of free medicine and diagnostic facilities have impacted positively on the patient utilization rate in the state of Rajasthan. Increased coverage of health insurance can add an extra protection from the health risks and early detection of disease conditions may help in achieving good health and lower treatment cost. On the eve of the epidemiological transition, rising share of non-communicable diseases will demand for facilities with diagnostic services (19, 20). So, time has come to change a move from quantity to quality.

## Author Contributions

Dr. DB is the main author responsible for the facts and figures. Dr. AT has assisted on shaping the ideas.

## Conflict of Interest Statement

The authors declare that the research was conducted in the absence of any commercial or financial relationships that could be construed as a potential conflict of interest.

## Footnotes

^1^Every 5 years, the Planning Commission (1) of India brings out a Plan document, detailing the budget outlays for and the areas of focus across various sectors of the economy. It is a development priority and direction road map.

^2^SC are those castes, which are part of the legislatively drawn schedule (Scheduled Castes), which in India benefit form reserved seats in public educational institutes, post schooling as well as in public jobs. Similarly, the ST (scheduled tribes) is a list of all those tribal groups, which are extended the same benefits.

^3^De-notified tribal groups are those which under the British occupation were legally termed as criminal tribes and were subsequently “de-notified” as non-criminal. They however continue to be associated with their past identity and face discrimination.

^4^Musahar is a low cast community who are extremely deprived and socially excluded and live on the margins of society.

^5^12th plan document, Chapter 2, p. 10.
